# Cytoplasmic DNA and AIM2 inflammasome in RA: where they come from and where they go?

**DOI:** 10.3389/fimmu.2024.1343325

**Published:** 2024-10-10

**Authors:** Conghui Xu, Weiyao Jing, Cui Liu, Bo Yuan, Xinghua Zhang, Limei Liu, Fengfan Zhang, Ping Chen, Qiang Liu, Haidong Wang, Xiaozheng Du

**Affiliations:** ^1^ Department of Acupuncture-Moxibustion and Tuina, Gansu University of Chinese Medicine, Lanzhou, China; ^2^ Department of Acupuncture and Pain, Affiliated Hospital of Gansu University of Traditional Chinese Medicine (TCM), Lanzhou, China; ^3^ Department of Acupuncture, Gansu Provincial Hospital of Traditional Chinese Medicine, Lanzhou, China; ^4^ Department of Zheng's Acupuncture, Affiliated Hospital of Gansu University of Traditional Chinese Medicine (TCM), Lanzhou, China; ^5^ Department of Rheumatic and Bone Disease, Gansu Provincial Hospital of Traditional Chinese Medicine (TCM), Lanzhou, China

**Keywords:** rheumatoid arthritis, AIM2, cytosolic DNA, pyroptosis, inflammasome

## Abstract

Rheumatoid arthritis is a chronic autoimmune disease of undetermined etiology characterized by symmetric synovitis with predominantly destructive and multiple joint inflammation. Cytoplasmic DNA sensors that recognize protein molecules that are not themselves or abnormal dsDNA fragments play an integral role in the generation and perpetuation of autoimmune diseases by activating different signaling pathways and triggering innate immune signaling pathways and host defenses. Among them, melanoma deficiency factor 2 (AIM2) recognizes damaged DNA and double-stranded DNA and binds to them to further assemble inflammasome, initiating the innate immune response and participating in the pathophysiological process of rheumatoid arthritis. In this article, we review the research progress on the source of cytoplasmic DNA, the mechanism of assembly and activation of AIM2 inflammasome, and the related roles of other cytoplasmic DNA sensors in rheumatoid arthritis.

## 1 Introduction

Rheumatoid arthritis (RA) is an autoimmune disease that manifests as persistent synovial inflammation of the joints, leading to proliferation of synovial tissue, erosion of articular cartilage and bone, and joint deformity and loss of function (1–3). RA can also cause multi-organ and multi-system damage, and patients are at higher risk than the general population for serious infections, respiratory disease, osteoporosis, cardiovascular disease, cancer, and death (3, 4). The cause of RA has not yet been fully characterized. The etiology of RA has not yet been fully elucidated, and studies have shown that a combination of factors contribute to the development of RA, such as smoking, air pollutants, diet, obesity, infections, microbiota, and genetic factors (5, 6).

During the pathogenesis of RA, cells of the immune system (e.g., macrophages, dendritic cells, etc.) recognize and take up foreign or self-antigens in the joints, such as bacteria or self DNA, which upon entry into the cells, can trigger the activation of cytoplasmic DNA sensors. Activation of cytoplasmic DNA sensors (cGAS, IFI16, etc.) leads to transcriptional up-regulation of type I interferon (IFN), pro-inflammatory cytokines, and other host defense genes, assembly of macromolecular signaling mechanisms, and induction of programmed cell death (7).

Pro-inflammatory cytokines such as TNF α, IL-1, and IL- 17 play a crucial role in the pathophysiologic process of RA, and they stimulate inflammation and degradation of bone and cartilage (8). Inflammasomes are one of the main sources of pro-inflammatory cytokines, which can be activated in response to injury or stress and participate in the defense mechanisms of intrinsic immunity (9). Melanoma deficiency factor 2 (AIM2) is one of the pattern recognition receptors (PRRs) that make up inflammasome, can bind directly to microbial dsDNA and its own dsDNA in a sequence-independent manner in the cytoplasm, initiating self-assembly and promoting the maturation of IL-1β and IL-18, mediating inflammatory responses (10).

Studies have shown that certain pathological alterations can lead to leakage of auto-DNA from the nucleus or mitochondria and accumulation in the cytoplasm, triggering an autoimmune inflammatory response (11). The nuclear membrane (NE) is the central organizing unit of eukaryotic cells, and by virtue of its highly selective, semi-permeable barrier function, the NE shields enclosed genetic material while ensuring its regulated transcription, replication, and repair (12). Consequently, the loss of nuclear membrane integrity results in the exchange of material between the nucleus and cytoplasm, facilitating the release of DNA from the cytoplasm. In addition, mitochondria, as semiautonomous organelles, contain their own genome (mtDNA), which is released into the cytoplasm when mitochondria are damaged in a stress response and functions as a damage-associated molecular pattern (DAMP). This paper reviews the source of DNA in the cytoplasm under pathological conditions, the mechanism of assembly and activation of AIM2 inflammasome, and its expression and function with other cytoplasmic DNA sensors in RA, aiming to provide some insights for the diagnosis and treatment of RA as well as the study of AIM2 inflammasome.

## 2 Source of cytoplasmic DNA

The cell’s own DNA is located primarily in the nucleus and mitochondria in interphase (13). Under normal conditions, there is no DNA in the cytoplasm. However, in the case of germs or viruses infecting the cell, or in the event of cellular damage, the DNA enters the cytoplasm. It is then recognized by cytoplasmic DNA receptors, which induce the cell to generate an intrinsic immune response through intracellular signaling pathways (14). Given that the multiple sources of cytoplasmic DNA have been described in detail in other articles, and in light of current research on RA, only micronuclei, mitochondrial DNA, and NETosis will be briefly described here (**Table 1**).

**Table 1:** Cytoplasmic DNA sources that may bind to AIM2.

Cytoplasmic DNA Source	Formation Pathway	References
Micronuclei	Chromosome lag	(15–17)
	The nucleus and NE fail to assemble correctly at mitotic exit	(18–20)
	Acentric chromosome segments are not integrated into daughter nuclei but are encapsulated in separate (often abnormal) NE	
Mitochondrial DNA	TFAM(mitochondrial transcription factor A) deletion	(21)
	VDAC oligomerizes under oxidative stress conditions	(22)
	Apoptosis	(23)
	Dysregulation of mitochondrial autophagy	(24–26)
	mPTP opening	(27, 28)
Suicidal NETosis	nuclear membrane rupture	(29, 30)
Vital NETosis	Nuclear DNA wrapped in vesicles released into the cytoplasm	(31, 32)

### 2.1 Micronuclei

Micronuclei (MN) are intact chromosomes or chromosome fragments encased in the nuclear membrane in the cytoplasm of the cell, separate from the nucleus, and generally produced by mitotic defects (33). Micronuclei form as a result of chromosome lag (15–17). They can also arise from the failure of the nucleus and nuclear envelope (NE) to assemble correctly at mitotic exit, as well as the failure of mitogen-free chromosome segments to integrate into the daughter nucleus, leading to their encapsulation in a separate (and often abnormal) NE (18–20). In addition, under sublethal stress conditions, a few mitochondria can be permeabilized through stress regulation, a process termed few MOMP. This results in limited cysteine asparaginase activation, which is insufficient to trigger cell death but causes DNA damage. This DNA damage, in turn, promotes genome destabilization and transformation, leading to a significant increase in the number of micronuclei (34).

MN is an extranuclear body, which unlike the primordial nucleus, is a double-stranded DNA fragment encapsulated in the nuclear membrane and present in the cytoplasmic lysate of the interphase (35). The nuclear membrane of MN is highly susceptible to spontaneous and irreparable rupture (35). The nuclear membrane is a barrier that protects the genome from the cytoplasmic environment and mediates proper nucleoplasmic translocation. Impaired integrity of the micronuclear envelope delays or disrupts DNA replication, inhibits DNA repair, and exposes micronuclear dsDNA directly to the cytoplasm (36). At this point DNA can be recognized by sensor proteins, which activate innate immune signaling pathways. Nuclear rupture in the micronucleus initiates a cGAS-dependent type I interferon response (37) and activation of AIM2 inflammasome (33, 38). As in Chen et al. (39), local disruption of the nuclear membrane induced by tumour treatment fields (TTFields) resulted in the release of a large number of micronuclei clusters into the cytoplasm, which strongly recruited and activated the two major DNA sensors cGAS and AIM2 and their corresponding cGAS/STING and AIM2/Caspase-1 inflammasomes, resulting in the production of pro-inflammatory cytokines, type I interferons (T1IFNs) and T1IFN-responsive genes (T1IRGs).MN is more common in patients and models of autoimmune and inflammatory diseases, suggesting that MN may be a source of immunostimulatory DNA (40).

MN can cause inflammation and inflammation can trigger MN (41). Inflammatory cells and mediators can disrupt the cellular genome through a variety of mechanisms. They can either directly induce DNA damage or directly or indirectly down-regulate DNA repair pathways and cell cycle checkpoints, destabilizing the cellular genome and contributing to the accumulation of MN (42). One study confirmed that peripheral lymphocyte MN frequency is increased in RA patients compared to healthy controls (43). MN frequency is also increased in buccal mucosal cells (44). This suggests that RA itself induces MN, however it is also possible that fragmented MN leads to DNA leakage into the cytoplasm, which triggers an innate autoimmune response and chronic inflammation. The production of MN has been linked to oxidative stress, DNA damage, and other factors.RA patients have elevated plasma levels of MDA, and reduced levels of GSH-Px and SOD, reflecting a higher degree of oxidative stress, a situation which may impair genetic stability in RA patients, as reflected by the fact that the genetic stability of RA patients is compromised. RA patients’ genetic stability, as reflected by an increased degree of DNA damage in RA patients and a positive correlation with plasma MDA levels in RA patients, and an increased frequency of MN (43). The steady accumulation of DNA damage in RA patients is a major contributor to the chronicity of the disease. And the steady accumulation of DNA damage increases chronic pro-inflammatory signaling and RONS production, increasing MN formation (45). Thus, MN may play a role in the pathogenesis of RA.

### 2.2 Mitochondrial DNA

Mitochondria play a vital role in the cell as the primary site of cellular respiration and energy production (46). Besides the nucleus, mitochondria are the only subcellular organelles in animals that contain the DNA genome (47). Mitochondria have a small amount of their own DNA known as mitochondrial DNA (mtDNA), which is a double-stranded circular molecule consisting of 16,569 base pairs that encodes a series of proteins critical for mitochondrial respiration (48, 49). Each mitochondrion contains one or more copies of mtDNA located in the mitochondrial matrix (50).

mtDNA and bacterial DNA share the same characteristics of conserved unmethylated CpG motifs (51). DNA containing unmethylated CpG motifs has been shown to have potent immunostimulatory effects (52). Therefore, mtDNA can act as DAMPs to activate specific receptors such as TLR9, AIM2, and cGAS to elicit chemokine production, thereby activating the immune response (53, 54). Which promotes the release of inflammatory mediators and the aggregation of inflammatory cells (55). The study showed that CIM2 and cGAS containing unmethylated Studies have shown that bacterial DNA containing unmethylated CpG motifs can induce arthritis (56). Collins et al. (57) found that the injection of mtDNA containing oxidatively damaged bases can trigger strong and persistent joint inflammation, with a few cases characterized by the formation of vascular opacities or bone destruction indicative of RA. Investigations of synovial fluid samples from patients also indicated that mtDNA is present in the majority of RA joints. This suggests that the level of mtDNA may be related to the extent of RA disease and may be a new diagnostic target for RA.

Genetic variation in mtDNA may also be associated with the pathogenesis of RA. Significantly increased frequency of SNPs 513 GCA > ACA, G at the RA mtDNA HVR-III locus and significantly increased frequency of SNPs in the HVRII region of individual RA patients compared to the HC region, suggesting their role in RA susceptibility (58). Further, the research findings of Zhang et al. indicate that the 16519C allele of the mtDNA D-loop might promote mtROS and IFN-γ levels by altering the replication and transcription of mtDNA, thereby modifying RA development (59). It also implied that improving the mitochondrial respiratory chain function might delay the occurrence of RA by reducing the level of mtROS.

Deletion of TFAM (mitochondrial transcription factor A) results in the release of mtDNA into the cytoplasm. TFAM is a transcription factor for mtDNA that determines the abundance of the mitochondrial genome by regulating packaging, stability, and replication, and plays an important role in maintaining the integrity of mtDNA as well as sustaining cellular functions (60, 61). TFAM deficiency leads to mitochondrial dysfunction (62). Mitochondrial dysfunction plays a role in RA by inducing a low-grade inflammatory response in RA-associated cells (e.g., synoviocytes) and increasing cellular sensitization and expression of cytokine-induced inflammatory mediators (63). It has been shown that TFAM is one of the major components in the formation of mitochondrial nucleoid structure, which can promote the complete compression of mtDNA into nucleoid to protect mtDNA from damage and degradation by the external environment by increasing the flexibility of DNA, facilitating the formation of DNA into loops, and exerting a U-shaped turn (64–66). TFAM deficiency promotes mitochondrial stress and mispackaging of mtDNAs, leading to their rejection into the cytoplasm (21), and recognized by cytoplasmic DNA sensors, thereby activating the innate immune response.

VDAC plays a role in mediating mtDNA release into the cytoplasm and inflammasome activation. Voltage-dependent anion channels (VDAC) are pore proteins located in the outer mitochondrial membrane that act as “gatekeepers” to control the entry and exit of metabolites, and play an important role in regulating the permeability of the outer mitochondrial membrane, maintaining mitochondrial homeostasis, and cell survival (67). When VDAC activity is inhibited, both mtROS generation and inflammasome activation are suppressed (68). VDAC can oligomerize under conditions of oxidative stress and VDAC oligomers can form large mitochondrial outer membrane pores (69). Thus oxidative stress mitochondria can release short mtDNA fragments through the pore formed by VDAC oligomers on the mitochondrial outer membrane (22). The VDAC oligomerization inhibitor VBIT-4 inhibits mtDNA release and reduces IFN signaling, neutrophil extracellular trap networks, and disease severity in a mouse model of systemic lupus erythematosus (22). mtDNA can be recognized by AIM2 (70), and mtROS are significantly associated with AIM2 activation and can be inhibited by antioxidant therapy (71).

It has been found that VDAC can be expressed in the plasma membrane of human osteoclasts and acts as a Cl^—^ channel to regulate the differentiation and function of human osteoclasts, and anti-VDAC antibodies inhibit the formation of human osteoclasts and bone resorption, which can be used for the treatment of diseases with increased bone resorption, such as RA (72). Increased osteoclast-mediated bone resorption can lead to localized or systemic bone loss, and its formation, differentiation, and activation in RA exacerbate bone destruction (73). VDAC opening increases mtROS production and causes oxidative stress, which in turn leads to mitochondrial dysfunction (74). Intracellular ROS up-regulate matrix metalloproteinases, which are involved in damaging the extracellular matrix, inducing cartilage degradation and an associated synovial inflammatory response that underlies the pathologic features of RA (75). Mitochondrial dysfunction can modulate innate immunity through redox-sensitive inflammatory pathways (e.g., NF-κB) or direct activation of inflammasome, and inflammasome activation in conjunction with the NF-κB pathway induces the expression of inflammatory cytokines, leading to a significant exacerbation of inflammatory responses (76). VDAC also recruits Parkin to defective mitochondria to promote mitochondrial autophagy (77), which prevents the accumulation of dysfunctional mitochondria and reduces oxidative stress and cell death, contributing to the suppression of the inflammatory response and alleviation of symptoms in RA patients.

The process of apoptosis also affects the accumulation of mtDNA in the cytoplasm. activated FLS in RA is characterized by hyperproliferation and resistance to apoptosis, and a lower-than-normal rate of apoptosis is thought to be the direct cause of synovial hyperplasia (78). Mitochondrial pathway-mediated apoptosis can control the proliferation of RA synoviocytes and the formation of vascular opacities (79). Bax-induced mitochondrial outer membrane permeabilization (MOMP) is considered to be one of the key control switches for apoptosis. Bax and Bak are direct pro-apoptotic effectors of MOMP because they can relocate and insert into the outer mitochondrial membrane to oligomerize and form large pores that release apoptotic factors such as cytochrome c (80). It was found that (23) after Bak/Bax activation and cytochrome c loss, the mitochondrial lattice ruptures. The Bak/Bax macropores that appear in the outer membrane provide an exit for the inner membrane, which carries mitochondrial matrix components, including the mitochondrial genome. This allows a portion of the protruding inner membrane to lose its integrity, exposing mtDNA to the cytoplasm. In addition, Cosentino et al. found that the relative effectiveness of BAX and BAK molecules determines the rate of growth of the apoptotic pore and its permissiveness to macromolecules, and affects the rate of release of mitochondrial content (especially mtDNA) during MOMP, with BAK-only-expressing cells (BAX^−/−^) releasing mtDNA much more rapidly than BAX-only-expressing cells (BAK1^−/−^) (81, 82).

Dysregulation of mitochondrial autophagy leads to mitochondrial damage, increased mtROS production, and mtDNA translocation into the cytoplasm, all of which are involved in the activation of inflammasome (83). Mitochondrial autophagy is the process by which mitochondria selectively remove damaged mitochondria through the autophagy mechanism to maintain normal mitochondrial function in response to external stimuli, and it is an important regulatory mechanism for mitochondrial quality control (84). It is an important regulatory mechanism for mitochondrial quality control. Deletion of the autophagy proteins Beclin 1 and LC3B, which are required for the early and late stages of autophagic vesicle formation, inhibits autophagy and disrupts mitochondrial homeostasis. This results in the accumulation of physiologically abnormal mitochondria, which stimulates excessive mtROS production and MPT. Consequently, this leads to the release of mtDNA into the cytoplasm (24). Ultimately, NLRP3 inflammasome and AIM2 inflammasome are activated. Tissue proteases and intracellular ROS released upon lysosomal rupture also severely affect mitochondrial membrane integrity, leading to membrane permeability and subsequent initiation of apoptosis (25, 26).

PINK1/Parkin-mediated mitochondrial autophagy is the classical mitochondrial autophagy pathway that has received the most attention and has been studied most extensively, and is closely related to RA progression. Gui Zhi Paeoniae Branches Granules (GSZG) promoted mitochondrial autophagy in osteoclast precursors and inhibited osteoclastogenesis by modulating the PINK1/Parkin pathway, thereby attenuating bone destruction and joint damage in CIA mice (85). Oxidative stress is a contributing factor in the pathogenesis of RA (75). Studies have demonstrated that oxidative stress can inhibit mitochondrial autophagy through down-regulation of the PINK 1/Parkin pathway, which promotes the abnormal proliferation of RA-FLS and contributes to the pathogenesis of RA (86–88). Tumor necrosis factor (TNF) is a key driver of RA, and TNF inhibits PINK 1-mediated mitophagy and leads to altered mitochondrial function and elevated cytoplasmic mtDNA levels (89). Compared with wild-type CAIA mice, PINK1^−/−^ mice showed slower progression of arthritis and significantly reduced swelling and inflammation (90). Additionally, PINK1 deletion in RA synovial fibroblasts (RASF) induced inhibition of mitochondrial autophagy and accumulation of damaged mitochondria. Furthermore, knockdown of PINK1 blocked RASF migration and invasion. Therefore, modulation of the PINK 1/Parkin-mediated mitochondrial autophagy pathway may be a potential therapeutic and diagnostic target for RA.

The opening of the mitochondrial permeability transition pore (mPTP) is associated with the release of mtDNA into the cytosol. mPTP is a non-selective, highly conductive channel spanning the gap between the inner and outer mitochondrial membranes. Classical structural modeling suggests that it is composed of a complex of multiple proteins, including VDAC, ANT, and CypD (91). The activation of mPTP leads to an increase in apoptosis, further exacerbating joint tissue damage and inflammation in RA. Mitochondrial Ca2 + uptake plays a key role in intracellular Ca2 + homeostasis. Excessive matrix Ca2 + concentrations, particularly when occurring in conjunction with oxidative stress, prompt the opening of mPTP, a mitochondrial inner membrane high conductance channel (92, 93). Under oxidative stress, mtDNA can be released via nonspecific mPTP. Under normal physiological conditions, transient opening of the pore in a subpopulation of mitochondria may also occur leading to the release of mtDNA fragments, whereas the likelihood of release of mtDNA via mPTP is significantly increased under pathological conditions (27). Frequent and prolonged activity of mPTP can lead to increased mtROS production and mitochondrial calcium overload, which further enhances mPTP opening. In addition, increased release of mtROS from mPTP exacerbates oxidative damage to nuclear DNA, leading to increased pro-apoptotic signaling, inducing the translocation of P53, p66sch, and other pro-apoptotic proteins to the mitochondria, where they enhance mPTP opening (28). The continuation of this cycle leads to increasingly weaker mitochondrial protective signals and stronger pro-apoptotic signals, ultimately leading to irreversible opening of mPTP, mitochondrial swelling, rupture of the outer membrane, and cell death (28).

### 2.3 Neutrophils extracellular trap (NETosis)

NETosis is a program for the formation of neutrophil extracellular trapping networks (NETs), a meshwork of dsDNA as the basic backbone, inlaid with a variety of protein molecules, which are wrapped in nuclear proteins, including histones, granule proteins (e.g., neutrophil elastase and myeloperoxidase), and cytoplasmic proteins (e.g., S100 calcium-binding proteins A8. A9 and A12, as well as actin and α-actinin) (94–96). NETs protect the host from infection by neutralizing and killing pathogens.

**Figure 1:**
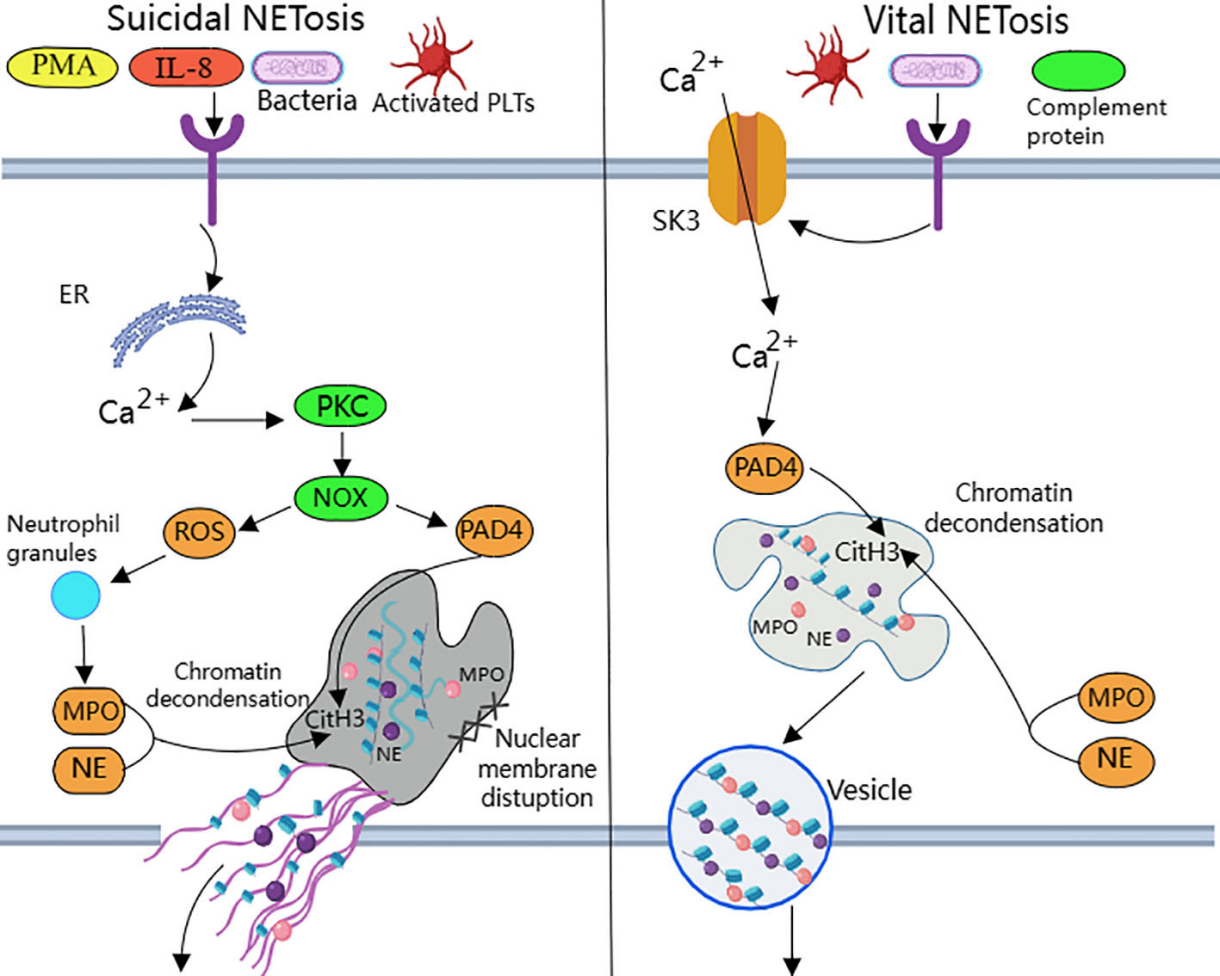
Suicidal and vital NETosis processes PMA, bacteria, cytokines, etc. can all induce ‘suicidal’ NETosis, when the receptor interacts with these stimuli, Ca^2+^ is released into the cytoplasm to activate PKC, leading to the production of ROS from NOX.ROS promote neutrophil granule ROS promote disassembly of neutrophil granules, resulting in the release of enzymes such as MPO and NE into the cytoplasm and subsequently into the nucleus. MPO, NE, and PAD4 together induce CitH3 citrullination, leading to chromatin derepression, followed by disassembly of the nuclear membrane, and mixing of decondensed chromatin with proteins released from cytoplasmic granules to form NETs. The other “life-type” NETosis consists of platelets, tonicity, and other proteins released by cytoplasmic granules. NETosis is activated by platelets, complement proteins, etc. After activation of neutrophils, Ca^2+^ is transferred to the cytoplasm via SK3, and the increased concentration of Ca^2+^ in the cytoplasm causes activation of PAD4, which leads to chromatin decondensation into the cytoplasm, and then participates in CitH3 citrullination and chromatin derepression in conjunction with MPO and NE, and finally, NETs are expelled through vesicles.

NETosis is categorized into suicide NETosis and vital NETosis, in both processes the release of nuclear DNA into the cytoplasm is an important step. Occurring in the presence of NADPH oxidase activity is suicide NETosis, which leads to neutrophil cell death (97). On the contrary, vital NETosis refers to a process in which neutrophils only squeeze out a small amount of DNA, preferentially squeeze out mtDNA, so that neutrophils can survive and continue to play an antibacterial role (98–100). PMA, bacteria, LPS, fungi, activated platelets, IL-8, IC, and numerous other stimuli can induce suicidal NETosis (101). In suicidal NETosis, after neutrophil activation, the intracellular calcium ion concentration increases, with Ca2+ being released from the endoplasmic reticulum into the cytoplasm, this activates protein kinase C (PKC) or the Raf-MEK-ERK MAP kinase pathway, leading to the production of ROS by the NADPH oxidase complex (NOX) and the activation of peptidylarginine deiminase 4 (PAD4) (29). PAD4 promotes citrullination of histone H3, which is necessary for chromatin dedensification, nuclear membrane disruption, and nuclear DNA release into the cytoplasm. ROS activate neutrophil elastase (NE), which enters the nucleus and subsequently begins to cleave histone, resulting in chromosome dedensification (102). Myeloperoxidase (MPO) is an enzyme downstream of NADPH oxidase. After being activated by intracellular ROS, MPO participates in chromatin decondensation and nuclear membrane rupture. It further activates NE, promoting NE transfer from the cytoplasm to the nucleus, which unfolds chromatin. Subsequently, the nuclear membrane disintegrates, and nuclear chromatin decompresses into the cytoplasm of intact cells, mixing with cytoplasmic and granular components. Eventually, the cell membrane ruptures, resulting in cell death and the release of NETs into the extracellular space (29, 30) (**Figure 1**).

Destruction of nuclear membrane is one of the most prominent features of NETosis. It was found that activation of cyclin-dependent kinase (CDK) plays an important role in signal transduction of NETs formation. CDK can pull neutrophils back from G0 phase to cell cycle, and silencing of CDK4 and CDK6 can block the release of NETs, and has no effect on intracellular ROS production, phagocytosis and degranulation (103). This indicates that cell cycle mechanism may be an important factor in promoting nuclear membrane decomposition during NETsosis. After nuclear membrane rupture, proteins released from cytoplasmic granules mix with decondensed chromatin to form NETs (104).

Mitochondria replace NOX as the source of ROS in vital NETosis (105). It requires extracellular calcium influx and is regulated by mtROS and small-conductance potassium channel 3(SK3) (106). TLR or some strains directly stimulate platelet binding to neutrophils causing PAD4 activation (100), chromatin decondensation is induced to enter the cytoplasm and participates in DNA densification in conjunction with MPO and NE (31). Chromatin that has been modified by proteins does not disrupt the cell membrane as it is excreted through nuclear membrane vesicles and vesicle outlets (32). The cell survives and remains functional. Considering that nuclear DNA encapsulated in vesicles is difficult to expose directly in the cytoplasm, the chance of recognition by pattern recognition receptors such as AIM2 may be reduced.

In some studies, AIM2 recognizes DNA in NETs and activates inflammasomes, possibly because many NETs consist of a DNA backbone derived from neutrophil nuclear DNA. According to Zeng et al. (107), NETs can be internalized into the cytoplasm via an unknown receptor and bind AIM2 to drive the assembly of an AIM2-mediated multiprotein complex, the AIM2 PANoptosome.Li et al. (108) reported that NETs mediate AIM2 inflammasome activation via NET DNA, which directly promotes alveolar macrophage pyroptosis, whereas administration of an active caspase-1 inhibitor (Ac-YVAD-cmk), a NET degrader (DNase I), and an inhibitor of NET formation (BB-Cl-amidine) prevented alveolar macrophage pyroptosis by promoting NET DNA degradation. A similar effect was achieved when AIM2 gene expression was silenced. This suggests a correlation between the release of NETs and AIM2 inflammasome-mediated pyroptosis. As shown by Antiochos et al. (109) that in SLE patients both AIM2 and IFI16 bind NETs, and the co-localization of AIM2 and IFI16 along NET chromatin fibers can be seen by confocal microscopy, suggesting that these two ALRs assemble into filaments on NET DNA. This ALR-NET structure protects the NETs from DNase I degradation, indicating that extracellular ALR-NET interactions may promote a mechanism for sustained IFN-I signaling. However, the ability to protect dsDNA from nuclease does not appear to be a universal property of all DNA-binding proteins, and cGAS does not share this ability.

NETs are the source of citrullinated antigens, and ACPA induces NETosis. Reticular neutrophils can be observed in peripheral blood, synovial fluid, synovial tissue, rheumatoid nodules and skin of RA patients (110). NETosis is enhanced in peripheral blood and SF neutrophils of RA patients, which is related to the presence of ACPA and the level of systemic inflammatory markers (110). The formation of NETs depends heavily on histone citrullination, which accounts for about 70% of all NETs proteins, it is known that PADs activated during the formation of NETs can citrullinate histone proteins (111). In addition, both the perforin and the membrane attack complex (MAC) pathways lead to the formation of pores in neutrophil membranes, inducing significant calcium influx and promoting the activity of the PAD enzyme. Thus, the perforin and MAC pathways activate intracellular PADs and induce hypercitrullination, which is activated in RA joints and can result in the production of ACPA (112). ACPA also externalizes citrullinated antigens (especially citrullinated histones), which are then targeted by ACPA to induce more NETosis (113). Furthermore, Khandpur et al. observed that NETs significantly enhanced the inflammatory response in RA and osteoarthritic (OA) synovial fibroblasts, including the induction of IL-6, IL-8, chemokines and adhesion molecules, and in turn, the inflammatory factors IL-17A, IL-18, and TNF-a induced NETosis in RA neutrophils (110). This process may be a vicious cycle develops in susceptible populations, leading to a continuation of the inflammatory response.

Studies suggest that the imbalance between NETosis and NETs degradation is closely related to the development and inflammatory process of RA (114). NETosis is enhanced in RA, and NETosis-derived products such as free DNA, free elastase, free nucleosomes, NE proteins, and MPO proteins are increased in the serum of RA patients (115). NETs are mainly degraded by DNaseI and phagocytosed by macrophages *in vivo*. If NETs are not effectively degraded, the abnormal formation of ACPAs is aggravated, which will inevitably further induce inflammatory responses and worsen joint inflammation. Spengler et al. found that during the pathogenesis of RA, the activity of DNaseI is reduced, serum free DNA levels are increased, a large number of autoantigens are exposed, and a large number of NETs are formed in the joint fluid. fluid with the formation of large numbers of NETs (116). Meng W et al. demonstrated that DNaseI degrades NETs in a concentration-dependent manner (103). Farrera et al. found that human monocyte-derived macrophages were able to phagocytose NETs in a cytosolic relaxin-dependent manner, and that both recombinant C1q and endogenous C1q extracted from human serum were able to condition NETs and promote their clearance (117).

Bone erosion in RA is primarily caused by synovitis, a process that involves activation of pro-inflammatory cytokines and activation of RANKL as well as production of antibodies against citrullinated proteins (118). Thus in addition to inflammation, anti-citrullinated protein (ACPA) antibodies are a major risk factor for the development of bone erosive disease in RA patients (119). ACPA binds directly to osteoclasts and stimulates osteoclast production, leading to bone destruction. This effect is based on the binding of ACPA to the surface of osteoclast precursors, which increases the number of osteoclast precursors by stimulating the production of tumor necrosis factor alpha (TNF-α) (120). ACPA-positive (especially antiporterin) patients with primary RA have higher serum and synovial RANKL concentrations, even after accounting for other inflammatory parameters, suggesting a direct link between ACPA and bone loss (121). Infusion of ACPA alone does not induce arthritis, but injection of ACPA after producing mild synovial inflammation significantly enhances the development and severity of inflammatory arthritis (122, 123). Thus, Inhibiting the production of NETs can effectively reduce synovial inflammation and alleviate arthritis (124). The results of animal experiments suggest that rhodopsin can improve RA by promoting neutrophil apoptosis and inhibiting NETosis (125).

## 3 Cytoplasmic DNA sensor

Cytoplasmic DNA sensors, a class of protein molecules that sense the presence of DNA in the cytoplasm, are able to rapidly and nonspecifically recognize extracellular danger signals and some intracellular self or non-self components, thereby activating the host immune system to generate an immune response against invading pathogens, apoptotic or damaged tissue cells. However, such indiscriminate DNA binding may lead to aberrant activation of cytoplasmic DNA sensors, triggering persistent or chronic inflammatory signals that promote the development of RA. Therefore, we believe that cytoplasmic DNA sensors play an important role in the pathogenesis of RA. The study of cytoplasmic DNA sensors and their regulatory mechanisms can help to gain insights into the pathogenesis of RA and provide new ideas and approaches for the development of relevant therapeutic strategies.

### 3.1 cGAS

cGAS was first proposed as a “cytoplasmic DNA sensor” concept (126). However, the subcellular localization of cGAS is not limited to the cytoplasm, but can also be found in the nucleus or anchored to the cell membrane. Similar to AIM2, the recognition of dsDNA by cGAS is sequence-unspecific and length-dependent. cGAS strongly catalyzes the reaction at very low concentrations of long DNA (>45 bp), whereas shorter DNA (~20 bp) does not activate cGAS efficiently upon binding (127). The molecule binds directly to double-stranded DNA and then catalyzes the production of the second messenger 2’3’ loop GMP-AMP (cGAMP) (128), which subsequently binds to STING localized in the endoplasmic reticulum, initiating the phosphorylation of TBK1 and IRF3 and activating NF-kB (129, 130). Activation of this signaling cascade promotes the expression of type I interferon and other immune-related cytokines. Whereas IFN-1 is an important cytokine in the innate immune response, its overexpression can cause autoimmune diseases (131). In addition, cytoplasmic DNA can trigger the AIM2 inflammasome signaling pathway via cGAS-STING, suggesting that cytoplasmic DNA may be involved in multiple parallel pathways whose cooperation contributes to maximizing inflammatory responses (132).

It has been noted that cytoplasmic dsDNA expression is increased in fibroblast-like synoviocytes (FLS) from RA patients and that the expression of dsDNA and cGAS correlates with the severity of RA synovitis (133). Additionally, cytokine expression is reduced in cells from RA patients with knockdown of cGAS or STING, which suggests that the cGAS-STING signaling pathway mediates the inflammatory response in RA (133). In Wang et al. study, cGAS was overexpressed in RA-FLS compared to OA FLS. cGAS overexpression significantly promoted the proliferation of RA FLS and enhanced AKT and ERK phosphorylation as well as the production of pro-inflammatory cytokines and matrix metalloproteinase (MMP) in TNF α-stimulated FLS (134). Animal results showed that down-regulation of AKT activation exerted anti-proliferative and anti-inflammatory effects in RA FLS and adjuvant arthritis (AIA) mice arthritis (135). Another study showed that antagonizing the PI3K/AKT signaling cascade inhibited RA synovial tissue angiogenesis and significantly reduced joint swelling and cartilage destruction in collagen-induced arthritis (CIA) mice (136). In addition, ERK was constitutively expressed in RA synovium and the activated phosphorylated form was much higher in RA synovium than in OA patients (137). The ERK signaling cascade promotes the production of inflammatory cytokines and MMPs. It is involved in synovial cell hyperproliferation and apoptosis inhibition, and also regulates osteoclast differentiation, activity, and chemotaxis. These actions exacerbate RA synovial inflammation and bone destruction (138–140). The ERK inhibitor FR180204 inhibits B cell activation and anti-CII Ab production as well as significantly reduces clinical arthritis scores in CIA rats and induces recovery from weight loss in this model (141). The above studies suggest that targeting cGAS to inhibit ERK and AKT pathways may have therapeutic potential in RA.

Li et al. (142) showed that cytoplasmic dsDNA-induced activation of cGAS/STING induced mtROS production, leading to MST1 phosphorylation and subsequent FOXO1 phosphorylation and nuclear translocation in RA FLSs, which enhanced FOXO1 transcriptional regulation of polarity- and migration-related genes and facilitated *in vitro* migration and invasion of RA FLSs. A study by Gu et al. (143) found that the expression of DNA polymerase β (Pol β), a key enzyme in base excision repair, was significantly reduced in peripheral blood mononuclear cells (PBMCs) from patients with active RA and CIA mice. Polβ deficiency led to the accumulation of DNA damage and leakage of cytoplasmic dsDNA. This triggered the activation of the cGAS-STING-NF-κB signaling pathway and resulted in the up-regulation of NLRP3, IL-1β, and IL-18 expression. Consequently, this exacerbated macrophage focal death and disease severity in CIA mice. In a study by Weng et al (144), macrophage extracellular traps (METs) were found to promote proliferation, migration, invasion, and high expression of inflammatory cytokines such as TNF, IL-1β, and the matrix degrading enzymes MMP-9 and MMP-13 in RA-FLS through activation of the cGAS-mediated PI3K/Akt signaling pathway. Willemsen et al. (89) demonstrated that cGAS deficiency blocked the interferon response and reduced inflammatory cell infiltration and joint swelling in a mouse model of inflammatory arthritis.

In summary, aberrant activation of cGAS is an important factor driving the onset and progression of RA, and therefore the development of inhibitors targeting cGAS will be helpful in the prevention and treatment of RA. Zhou et al. (145) To inhibit the excessive pro-inflammatory response associated with cGAS-STING overactivation in some autoimmune diseases, nanomedicinal hydrogels (NiH) were designed, which co-delivered the cGAS inhibitor RU.521 (RU) and cationic nanoparticles (cNPs) that scavenge free DNA (cfDNA) to draining lymph nodes (LNs), for systemic RA immunotherapy for Immunosuppression. The expression of cGAS has been reported to be upregulated in RA patients and CIA mice, and subcutaneous administration of NiH delayed the progression of RA and reduced the severity of arthritis in a CIA mouse model (146). It is well known that M1 macrophages are the main cellular phenotype of RA-activated macrophages. The study by Xu et al. designed FDL@TP nanomicelles, which achieved active targeting of articular M1 macrophages, significantly down-regulated the expression of cGAS and STING proteins, and inhibited the secretion of TNF-α, IL-1β, and IL-6 by M1 macrophages, thus exerting their anti-inflammatory and immunosuppressive effect, reducing joint inflammation and pain symptoms in AIA mice (147).

### 3.2 IFI16

IFI16 belongs to the PYHIN family of proteins along with AIM2, which consists of an N-terminal PYRIN structural domain (PYD), and one or two C-terminal HIN-200 structural domains. Among them, the PYD structural domain is involved in homotypic protein-protein interactions, while the HIN structural domain recognizes dsDNA and ssDNA (148). Unlike AIM2, the C-terminus of IFI16 contains two HIN structural domains called HIN- A and HIN- B (132). Where mutations in the HIN-B structural domain lead to decreased IFN-β production, mutants in the HIN-A structural domain increase IFN-β production. The detection of DNA in the cytoplasm leading to IFN-β induction has been suggested to be a trigger for autoimmune disorders, thus providing further evidence that the detection of DNA by IFI16 may play a role not only in the antiviral innate immune response but also in the response to bacterial pathogens and autoimmune responses as well (149, 150).

IFI16 exhibits both nuclear and cytoplasmic localization. It has been reported that when IFI16 protein senses cytoplasmic dsDNA, it recruits the interferon gene stimulating factor (STING) protein, which promotes IFN-β expression by activating the transcriptional activities of IRF3 and NF-κB (149). The study by Kerur et al. (150) that when endothelial cells are infected with Kaposi’s sarcoma-associated herpesvirus (KSHV), the IFI16 protein interacts with ASC and pro-caspase-1 to form functional inflammasome, a complex that is initially located in the nucleus and then further translocates to the cytoplasm and leads to the activation of caspase-1 and the cleavage of pro-IL-1β and pro-IL-18 to their mature forms. In addition, IFI16 can heterodimerize with AIM2 and studies have confirmed that the IFI16 protein negatively regulates the activation of caspase-1 by the AIM2-ASC inflammasome and upregulates the expression of IL-10, exerting an anti-inflammatory effect (151).

IFI16 protein has been found to be present in the serum of many patients with autoimmune diseases, including systemic sclerosis (SSc), systemic lupus erythematosus (SLE), desiccation syndrome (SjS), and RA.It is noteworthy that the highest serum IFI16 protein levels were found in a limited number of RA subjects (152). A study by Alunno et al. Found that (153) both serum IFI16 and anti-IFI16 antibody levels were higher in RA patients than in HC patients, and IFI16 concentrations were directly correlated with anti-IFI16 antibody titers. The majority of RA patients with detectable circulating IFI16 protein were also positive for RF/ACPA, and the presence of circulating IFI16 protein was significantly associated with RA-associated lung disease. Mean synovial fluid (SF) concentrations of both IFI16 protein and anti-IFI16 antibodies were higher in RA than in control OA. These data suggest that IFI16 may be present as a circulating protein in serum of patients with RA and participates in inflammatory processes in target tissues, but its relevance as a new clinical biomarker for RA needs to be confirmed by further studies. Gugliesi et al. observed that IFI16 can be overexpressed in response to inflammatory stimuli and then released in the extracellular milieu, binding to endothelial cells and causing damage, suggesting that this protein has novel pathogenic and alerting functions and an important contribution to the development of chronic inflammation and autoimmunity (152). This is corroborated by Bawadekar et al. (154), who showed that extracellular IFI16 protein acts as a DAMP, transmitting a ‘danger signal’ that induces endothelial cells to produce IL-6, IL-8, CCL2, and CCL5. This process occurs through the activation of the p38 MAPK and NF-κB p65 signaling pathways, along with CCL20 and other pro-inflammatory cytokines.

These above findings imply that IFI16 may be involved in the pathophysiology of autoimmune diseases such as RA, and that modulation of IFI16 may be a novel therapeutic strategy for it. For example, in the study of Li et al. (155), STING prevented autoimmune diseases by recruiting the E3 ligase TRIM 21 to promote IFI16 degradation via the ubiquitin-proteasome pathway in order to maintain the appropriate IFI16 protein level and prevent host cells from overproducing IFN-I during antiviral innate immunity.

### 3.3 ZBP1

ZBP1, also known as DAI (DNA-dependent activator of IFN regulatory factors), was the first cytoplasmic DNA sensor to be discovered and was shown to directly bind dsDNA in mouse L929 fibroblast-like cells (156). ZBP1 contains two N-terminal Z-DNA-binding domains (ZBDs, termed Zα1 and Zα2), two RIP homotypic interaction motif structural domains (RHIM1 and RHIM2) and a C-terminal signaling domain (SD) (157, 158). Whereas ZBD senses Z-type or other types of nucleic acid ligands, binding of ZBP1 to dsDNA requires the D3 structural domain (a region that overlaps with the RHIM1 structural domain), which functions under the assumption that the initial binding of the D3 region to the DNA results in a change in protein conformation that allows access to the other two Z-DNA binding domains (159). In addition, the dsDNA ligand for ZBP1 is not sequence specific, but longer dsDNA fragments are more likely to activate ZBP1 (159). ZBP1 also plays an important role in the immune monitoring of mtDNA. The instability of the mitochondrial genome promotes the accumulation of Z-type DNA, whereas ZBP1 is able to stabilize Z-type mtDNA and form a complex with other immune-related proteins, such as cGAS, RIPK1 and RIPK3, which together maintain IFN-I signalling and participate in the regulation of the innate immune response (160).

ZBP1 binds to receptor-interacting protein kinase 3 (RIPK3) via the RHIM structural domain and promotes the autophosphorylation of RIPK3 (161). It also induces the phosphorylation of the downstream necroptotic apoptosis enforcer mixed linear kinase structural domain-like (MLKL), which leads to necroptotic apoptosis (161). ZBP1 also interacts with RIPK1 and RIPK3 and mediates the NF-κB signaling pathway dependent on their shared RHIM structural domains (157). The RIP1 inhibitor necrostatin-1 has been shown to reduce articular cartilage damage and necroinflammation in AA rats (162). The study by Jhun et al. (163) demonstrated that necrostatin-1 treatment decreased the expression of necrotic apoptotic mediators RIPK1, RIPK3, and MLKL. It also inhibited osteoclastogenesis, down-regulated Th1 and Th17 cell expression, promoted Th2 and Treg cell expression, and reduced pro-inflammatory cytokine production in the synovial membrane of CIA mice, thereby delaying CIA progression.

The SD structural domain of ZBP1 recruits TBK1 and IRF3 to activate type I IFN synthesis and other inflammatory responses (164). IFN-I is elevated in the serum of patients with autoimmune diseases such as SLE, RA, SSc and Sjögren syndrome (SS).Clinical trial data suggest that a high IFN-I profile can be used as a biomarker in high-risk individuals to predict RA (165). Another study found that ZBP1 binds pyrin, AIM2, ASC, caspase-1, caspase-8, RIPK1, RIPK3, and FADD to form large multiprotein complexes that drive PANoptosis and host defense (166). Whereas PANoptosis can be simultaneously involved in three key modes of programmed cell death, including pyroptosis, apoptosis, and necrosis. It is highly likely that it has a potential role in RA (167).

Taken together, ZBP1 plays a crucial role in host immunity against autoimmune diseases driven by its own nucleic acids. However, our current understanding of the interaction between ZBP1 and cytoplasmic auto DNA and its relationship with RA is still limited, and more experimental data are needed to fully understand the multiple functions of ZBP1 in it.

### 3.4 TLR9

Toll-like receptors (TLRs) are front-line sensors of danger signals released after injury or infection and by pathogens (168). The TLR consists of a leucine-rich repeat sequence (LRR), a transmembrane structural domain, and a cytoplasmic Toll/Interleukin-1 receptor homology (TIR) structural domain. The LRR of TLR9 is located on the inner side of the membrane compartment, while the TIR structural domain is located on the cytoplasmic side (169). TLR9 is localized in intracellular membrane compartments such as the endoplasmic reticulum (ER), nuclear endosomes and lysosomes, which recognizes internalized bacterial DNA and unmethylated CpG oligonucleotides (170). Ribosomal DNA containing unmethylated CpG repeats released from dead cells in RA serum may be an endogenous ligand for TLR9, which is implicated in the development of RA (171).

TLR9 is elevated before the onset of RA and its expression increases with disease progression (172). Studies have shown that inhibition of TLR9 prior to disease onset significantly reduces arthritis and almost completely eliminates bone erosion, and reduces serum IL-6, a-1-acid glycoprotein, and rheumatoid factor levels in PIA rats. Furthermore, TLR9 expression in precursor cells was higher than that in mature osteoclasts and osteoclastogenesis was partially inhibited only by TLR9 antagonists. These results confirm the pathogenic role of endogenous DNA and TLR9 in triggering the initiation of joint autoimmune responses (173). At the same time, the expression of TLR9 is increased on monocyte subpopulations in patients with active RA, and TLR agonists cause increased production of inflammatory cytokines (174). A Turkish study found a significant difference in the frequency of the rs187084 allele of the TLR9 gene between RA patients and controls (p=0.003), suggesting that polymorphisms in the TLR9 gene may be associated with the pathogenesis of RA (175).

The TIR structural domain is known to be the signaling structural domain of TLR9, and MyD88 is an adaptor protein that mediates the binding of the TLR9 TIR structural domain to downstream signaling molecules to activate inflammatory and immune responses. Oxidized Mucuna pruriens (OMT) significantly down-regulated the expression of TLR9, IL-21, MyD88, STAT3, p-STAT3, and CXCR5, while up-regulating the expression of Foxp3, Blimp-1, and CTLA-4 in the synovial tissues of CIA mice (176). This action maintained the immune balance between Tfr and Tfh cells by regulating the TLR9-MyD88-STAT3 signalling pathway, reducing joint swelling and arthritis scores, and improving joint injury in CIA mice (176). Xu et al. demonstrated that (177) ST3GAL3 overexpression upregulated the expression of proliferation-associated proteins (cyclin D, cyclin E, and proliferating cell nuclear antigen) and TLR pathway-enriched factors (TLR9 and MyD88). It also increased the production of MMP1, MMP3, IL-6, and IL-8. Notably, TLR9-specific siRNA reversed the effects of ST3GAL3 overexpression on the proliferation, migration, and inflammation of MH7A cells, which are a human RA-derived FLS cell line. Thus, ST3GAL3 may be involved in RA pathogenesis through the activation of the TLR9/MyD88 pathway.

TLR9 plays an important role in the pathologic process of RA, therefore, the development of new strategies that can inhibit TLR9 activation will help to enrich the therapeutic options for RA. Hydroxychloroquine (HCQ), one of the most commonly used immunosuppressants for the treatment of RA, has a protective effect against RA arthritis by inhibiting DC maturation and migration through blocking TLR9 (178). A study by Han et al. (178) showed that HCQ downregulated TLR9 expression not only in serum-stimulated healthy donor PBMC-derived DCs from RA patients, but also in LN DCs and CpG-activated BMDCs from CIA mice. Additionally, TLR9 knockout (TLR9^-/-^) mice exhibited lower arthritis scores and attenuated synovial destruction and inflammatory cell infiltration compared with wild-type (WT) mice, which had impaired DC maturation and migration. In a study by Torigoe et al. (179), HCQ, a TLR9 antagonist, inhibited CpG-induced IL-6 and TNF-α production in B cell subsets and significantly suppressed TLR9-mediated human B cell function during inflammation.

### 3.5 AIM2

#### 3.5.1 Assembly and activation of AIM2 inflammasome

AIM2 is a cytoplasmic sensor that recognizes double-stranded DNA of microbial or host origin (180). The ability of dsDNA to effectively activate AIM2 inflammasomes depends on its length and is independent of sequence. Biochemical studies have shown that 80 base pairs (BP) dsDNA is the minimum length required to activate AIM2 inflammasomes (181). This length specificity may be a protective mechanism acquired by humans during the evolutionary process. By limiting the response to short DNA fragments, human cells can avoid an excessive immune reaction to short DNA segments produced during normal physiological processes.AIM2 protein consists of PYD domain at N-terminal and HIN-200 domain at C-terminal (182). The C-terminal HIN structure has the ability to recognize dsDNA, while the N-terminal PYD domain has a strong tendency to self-polymerize and interacts with other PYD-containing proteins via PYD-PYD interactions (183). Under steady-state conditions, AIM2 exists in a self-inhibitory conformation, where the HIN domain interacts with the PYD domain to form an inhibitory state (10). In this state, the PYD domain cannot recruit ASC (10). However, the binding of the HIN domain to dsDNA can result in a conformational change that releases the PYD domain from self-inhibition to interact with the ASC (181).

The N-terminal PYD structural domain of AIM2 interacts via PYD-PYD and recruits the junction protein ASC (CARD-containing apoptosis-associated speck-like protein) (184). ASC acts as an articulatory protein involved in inflammasome assembly and apoptosis (185). ASC consists of two death domains, the pyrin structural domain (PYD) located at the N-terminal end and the caspase recruitment structural domain (CARD) located at the C-terminal end. ASC recruits the effector protein pro-Caspase-1 through CARD-CARD interactions, assembles to form AIM2 inflammasome, and induces pro-Caspase-1 auto processing to produce activated Caspase-1 (186). Activated Caspase-1 cleaves the immature forms of pro-inflammatory cytokines pro-IL-1β and pro-IL-18 to the mature forms IL-1β and IL-18 (187). Caspase-1 also cleaves the GSDMD protein (a protein involved in cytolysis and lysis), removing its C-terminal fragment and releasing the N-terminal fragment to form large pores in the plasma membrane, leading to loss of cell membrane integrity and release of mature IL-β and IL-18 (188). causing cellular pyroptosis. This process is defined as the classical pathway of AIM2 activation.

**Figure 2:**
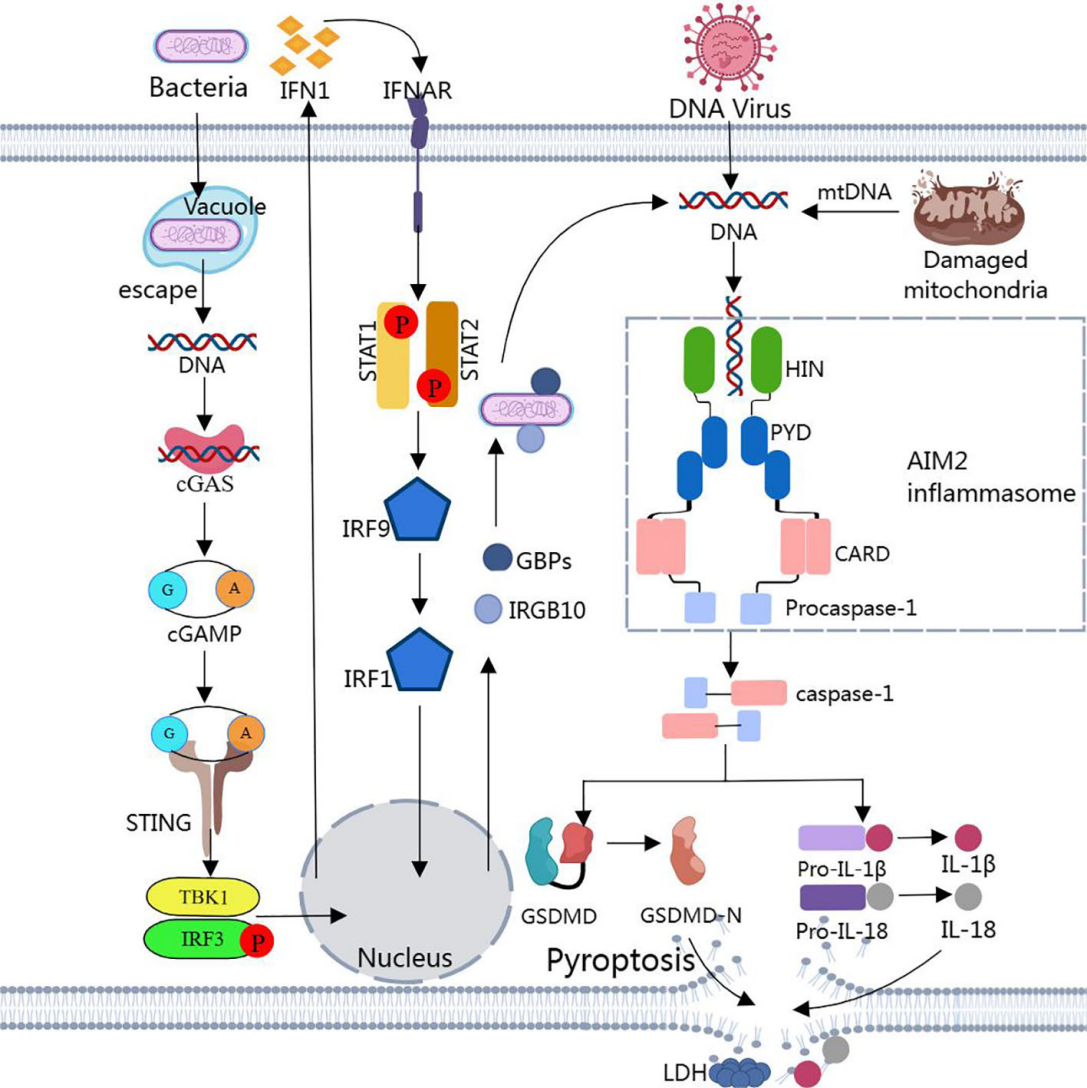
Assembly and activation of AIM2 inflammasome Binding of dsDNA released from bacteria, viruses, or damaged mitochondria to AIM2 triggers the assembly of AIM2 inflammasome. Activated caspase-1 cleaves pro-IL-1β, pro-IL-18, and GSDMD to produce IL-1β, IL-18, and GSDMD-N. GSDMD-N inserts into the lipid structure of the cell membrane to form a plasma membrane pore, causing cellular pyroptosis. Notably, cytoplasmic bacteria activate AIM2 inflammasome via a type I interferon-dependent “non-classical” pathway, and GBPs and GTPase-mediated lysis results in the release of large amounts of bacterial dsDNA into the cytoplasm for AIM2 recognition.

However, most bacteria that enter the host cytoplasm are usually encapsulated by the plasma membrane, and only a small number of bacteria are directly exposed to the host cytoplasm and release dsDNA. Due to the low concentration of dsDNA, the amount of dsDNA in the cell is not sufficient to activate AIM2, and then non-classical pathways are required to induce the production of a larger amount of dsDNA in the cell to activate AIM2 (189, 190). Through other cytoplasmic sensors of dsDNA, such as cyclic GMP-AMP synthase (cGAS) and IFN-inducible protein IFI204, the production of type I interferons is induced (183, 191). Type I IFN is secreted extracellularly and binds to type I interferon receptors (IFNAR) in an autocrine manner (183, 191). The binding of IFNAR with interferon regulatory factors IRF9 and IRF1 stimulates the expression of guanylate binding proteins (GBPs) and immune-related GTPase-family member b10 (IRGB10) (183, 191). These factors synergistically trigger bacterial lysis, releasing substantial amounts of dsDNA into the cytoplasm at adequate concentrations for AIM2 detection and recognition. This leads to the reactivation of AIM2 via the classical pathway (183, 191) (**Figure 2**).

#### 3.5.2 Negative regulation of AIM2 inflammasome

Under normal conditions, the immune system has a strong self-regulatory capacity and a series of negative feedback mechanisms to inhibit the activation of the AIM2 inflammasome and thereby modulate the inflammatory response, e.g., by blocking caspase-1 activation and inhibiting ASC phosphorylation. These different regulatory systems ensure that the appropriate level of inflammation is initiated to counteract cellular damage while protecting tissue structure. Since the assembly of AIM2 inflammasome relies on PYD- and CARD-mediated protein interactions for nucleation and maintenance of protein aggregation. Therefore, PYD-only proteins (POPs) containing the PYRIN structural domain (PYD) and CARD-only proteins (COPs) containing the caspase recruitment structural domain (CARD) as small endogenous inhibitory proteins provide a unique competitive binding mechanism capable of disrupting the assembly of the AIM2 inflammasome by competing for binding sites. Furthermore, it was demonstrated that interfering with key interactions reduced the local density of essential components of inflammasome and raised the threshold for inflammasome activation (192).

Three POP genes have been identified in the human genome: POP1 (also known as ASC2), POP2, and POP3. POP1 and POP2 can both broadly inhibit typical inflammasome responses by binding to ASC, preventing sensor-induced ASC oligomerization, disrupting the assembly of inflammasomes (193). ASC particles have been reported to be present in the serum of patients and mice with autoimmune and autoinflammatory diseases (194). Whereas POP1 not only prevents the assembly of inflammasome and the release of cytokines, it also prevents the release of ASC dangerous particles and inactivates ASC particles by doping them with POP1, thus preventing the self-perpetuation of inflammasome responses in neighboring cells (195). Stehlik et al. demonstrated that transgenic POP1 expression protects mice from systemic inflammation (195). Compare to POP1 and POP2, POP3 has high similarity to the PYD sequence of AIM2 and contains multiple HIN-200 PYD-specific sequence motifs, which are able to bind directly to the PYD structural domain of AIM2, preventing AIM2 from interacting with the ASC, thereby disrupting the assembly of AIM2 inflammasome and inhibiting the release of IL-1β, IL-18 (196).

COP/Pseudo-ICE (CARD16), INCA (CARD17) and ICEBERG (CARD18) are the first subgroup of COPs (197). Due to their high sequence homology with the Caspase-1 CARD domain, they are considered as the primary negative regulators of Caspase-1 activation (198). ICEBERG has a high degree of structural similarity to the CARD of Caspase-1 (53% protein sequence homology) and isolates Caspase-1 through CARD interactions, thus functioning as a decoy protein and preventing it from binding to activating aptamers. Pseudo-ICE has 92% sequence homology to the Caspase-1 CARD protein (199). Similar to ICEBERG, Pseudo-ICE interacts with the CARD structural domain of Caspase-1 to prevent Caspase-1 activation, thereby inhibiting the assembly of inflammasome and the inflammatory response (200). INCA has 83% homology to Caspase-1 CARD (192). Its mechanism of action is to specifically bind to the filamentous form of Caspase-1, localize at the tip of Caspase-1 CARD filaments, act as a cap, and prevent Caspase-1 CARD polymerization and subsequent inflammasome activity at very low concentrations (192).

Autophagy can mediate the degradation of AIM2 inflammasome to terminate the inflammatory response. Shi et al. (201) reported that the induction of the AIM2 inflammasome in macrophages triggers the activation of the G protein RalB and the formation of autophagosomes. Furthermore, the induction of autophagy is not dependent on ASC or caspase-1, but rather on the presence of inflammasome sensors (201). The assembled inflammasome undergoes ubiquitination and recruits the autophagy adapter p62. By virtue of its LC3-binding domain, p62 can deliver the inflammasome into the autophagosome for degradation, thus limiting the activity of the inflammasome (201). Rodrigue-Gervais et al. (202) showed that the mitochondrial serine protease HtrA2 prevents the long-term accumulation of the inflammasome articulating protein ASC by up-regulating autophagy, thus limiting AIM2 inflammasome activation. Following DNA virus infection, tripartite motif 11 (TRIM11) binds to AIM2 through its PS structural domain and undergoes auto-ubiquitination at the K458 site (203). This process facilitates the association between TRIM11 and the autophagy cargo receptor p62, which mediates the degradation of AIM2 through selective autophagy (203). A study by Wang et al. confirmed (204) that dihydroartemisinin (DHA) is able to exert its therapeutic effects on cutaneous squamous cell carcinoma (cSCC) by promoting autophagy and thus inhibiting the activation of the AIM2 inflammasome pathway.

It is well known that a controlled and balanced inflammasome response is essential for maintaining homeostasis in the body. Through a series of complex biochemical reactions and signaling pathways, the negative regulatory mechanisms of the inflammasome form an effective feedback loop. This loop ensures the body’s rapid and effective defense against invading pathogens while avoiding potential damage caused by an overreaction of the autoimmune system.

#### 3.5.3 AIM2 inflammasome and cytoplasmic DNA

Activation of AIM2 inflammasome requires cytoplasmic DNA as a trigger. Cytoplasmic degradation nucleases such as TREX1 and DNase II, as well as the DNA repair nuclease MRE11A, are commonly impaired or absent in RA (205, 206). This deficiency leads to the accumulation of cytoplasmic DNA, which activates intracellular immune responses, including AIM2-mediated inflammatory responses. As demonstrated by Luo et al. (207) that local overexpression of TREX1 suppresses synovial inflammation in rats, whereas conditional genomic deletion of TREX1 in AIA rats results in abnormal immune activation. In a study by Li et al. (208), loss of MRE11A function leads to mitochondrial failure, reduced ATP production, and mtDNA leakage into the cytoplasm, triggering inflammasome activation and cellular pyroptosis, ultimately leading to tissue inflammation. In addition, defective MRE11A activity induces premature aging of T cells in RA, and MRE11A^low^ T cells differentiate into hyperactive and tissue-invasive effector cells, thereby promoting destructive synovitis (209). In summary, the enzymes TREX1, DNase II, and MRE11A each play an important role in maintaining intracellular DNA homeostasis and genomic integrity, which are not only critical for maintaining cellular health, but also have a profound impact on the course of RA. Changes in their expression levels in RA patients may be one of the ways in which RA spontaneously relieves inflammation. However, currently there is relatively little research on the upregulation pathways or drugs of these nucleases. Other studies show that supplementing with vitamin D can reduce the rate of RA disease onset, pain levels, and disease activity scores (210, 211). In addition, consuming fish/omega-3 fatty acids can inhibit leukocyte chemotaxis, adhesion molecule expression, and the production of pro-inflammatory leukotrienes and prostaglandins (212, 213). They also downregulate adhesion molecules on endothelial cells (such as VCAM-1, ICAM-1, E-selectin), thereby exerting anti-inflammatory effects. These effects, in turn, help alleviate arthritis inflammation and pain in RA patients (212, 213). However, there is still a lack of research on whether the mechanisms that mediate the resolution of RA inflammation are related to cytoplasmic DNA clearance or AIM2 activity regulation. Exploring the specific mechanisms of spontaneous resolution of RA inflammation can deepen people’s understanding of RA and provide new insights for the clinical treatment of RA.

AIM2 inflammasome not only promotes the development of RA, but also plays an important regulatory role in the pathogenesis of a variety of other autoimmune and inflammatory diseases such as psoriasis, SS, SLE, etc. A study by Vakrakou et al. (214) showed that the ductal salivary epithelium of SS patients exhibited significant accumulation of damaged genomic DNA in the cytoplasm, which co-localized with AIM2. Furthermore, DNase1 expression and activity were found to be significantly impaired in these patients, and this impairment negatively correlated with inflammatory vesicle activation. Notably, down-regulation of DNase1 by approximately 60% resulted in significant up-regulation of AIM2, ASC/PYCARD, and pro-IL-1β genes, as well as an increase in IL-1β production. This suggests that there is a defect in cytoplasmic DNA degradation in SS patient cells, and that the cell-intrinsic activation state of the ductal epithelium in SS patients is due to persistent activation of epithelial AIM2 as a result of aberrant accumulation of cytoplasmic DNA.

Dombrowski et al. (215) detected abundant cytoplasmic DNA and increased AIM2 expression in keratinocytes from psoriatic lesions, but not in healthy skin. In addition, findings showed that the antimicrobial peptide LL-37, which has antimicrobial properties, can interact with DNA in psoriatic skin and neutralize cytoplasmic DNA in keratinocytes, thereby blocking the activation of AIM2 inflammasome. EFLA 945 is a red grape leaf extract that can limit the activation of AIM2 inflammasome. Chung et al.’s study found (216) that EFLA 945 significantly inhibited apoptosis-associated speckled proteins triggered by the AIM2 agonist poly(dA:dT) (CARD(ASC)-containing oligomerization), caspase-1 activation, and secretion of IL-1β and IL-18 by blocking the entry of DNA into THP-1-derived macrophages and ameliorated the severity of psoriasis in an *in vivo* mouse model.

Monteith et al. (217) reported that macrophages from lupus-susceptible MRL/*lpr* mice failed to mature lysosomes, thereby promoting the accumulation of IgG immune complexes (IgG-ICs) containing apoptotic debris, which would promote phagolysosomal membrane permeabilization, allow leakage of dsDNA into the cytoplasm, activate AIM2, and trigger the assembly of inflammasome. Zhang et al. showed (218) that the expression level of AIM2 in apoptotic DNA (apopDNA)-induced lupus mice was positively correlated with the level of anti-dsDNA antibodies, suggesting that there is a correlation between the expression level of AIM2 and dsDNA in the pathogenesis of lupus nephritis. Ge et al. found (219) that Legionella DNA was released into the macrophage cytoplasm This effect was significantly enhanced by the deletion of the Dot/Icm translocation effector SdhA. The SdhA protein could block the AIM2 inflammasome-mediated innate immune response by participating in cell membrane translocation, maintaining the membrane integrity of Legionella bacterial endosomal vesicles (LCVs), and preventing the release of bacterial DNA into the cytoplasm of infected macrophages.

#### 3.5.4 Protective effects of AIM2

AIM2 is known to sense intracellular DNA of a wide range of microbial pathogens, initiating a natural immune response to help clear infections. Its inappropriate recognition of its own DNA in the cytoplasm may contribute to immune-related diseases such as psoriasis, arthritis, and SLE. However, AIM2 has also been shown to exhibit important protective roles in several physiological and pathological processes such as tumor immune surveillance and neurodevelopment.

AIM2 is involved in the DNA-dependent activation of the intestinal mucosal immune system and is a key regulator of intestinal homeostasis. Numerous studies have demonstrated ecological dysregulation in the gut of *Aim2* ^−/−^ mice.Hu et al. found that after DSS administration, more aerobic and anaerobic/parthenogenetic anaerobic bacteria were present in the colonic tissues of *Aim2* ^−/−^ mice compared to WT mice, and cultures of fecal homogenates showed a higher bacterial load in the colon of *Aim2* ^−/−^ mice (220). 16S rRNA gene sequencing analysis revealed significant differences in the diversity and composition of the intestinal flora between WT and *Aim2* ^−/−^ mice reared separately. Compared to WT mice, *Aim2* ^−/−^ mice harbored increased levels of Akkermansia muciniphila and Anaeroplasma, while exhibiting decreased levels of Anaerostipes, Bifidobacterium, Flexispira, Prevotella, and Paraprevotella species (221). Notably, the exchange of gut microbiota between *Aim2* ^−/−^ mice and healthy WT mice enhanced the susceptibility of WT mice to colitis and colorectal cancer (220, 221). Hu et al. also found that (220) that IL-1β and IL-18 produced by AIM2 inflammasome are involved in the regulation of gut microbiota such as E. coli by inducing antimicrobial peptides in intestinal epithelial cells. Ratsimandresy et al. also demonstrated that AIM2 inflammasome up-regulate antimicrobial peptide expression through the IL-18/IL-22/STAT3 signaling pathway in order to regulate the intestinal ecological homeostasis (222). Current scientific research suggests that intestinal inflammation and decreased barrier function promote the pathogenesis of clinical RA (223). Is there a correlation between this and the adjustment of gut flora by AIM2? We do not yet know.

AIM2 was originally identified as a tumor suppressor gene in melanoma (224). In different types of tumors, AIM2 exhibits either pro- or anti-tumorigenic effects. In colon, hepatocellular carcinoma (HCC), breast, renal carcinoma (RCC), and cervical cancer, decreased AIM2 expression promotes tumorigenesis and affects patient prognosis. In a study by Chen et al. (225), AIM2 protein expression was significantly reduced in HCC cell lines and clinical samples. This reduction in AIM2 was strongly associated with higher serum AFP levels, vascular infiltration, poor tumor differentiation, incomplete tumor envelope, and unfavorable odds of survival after surgery. Ma et al. found that exogenous overexpression of AIM2 inhibited the mammalian mTOR-S6K1 pathway, which in turn inhibited the proliferation, colony formation, and invasion of hepatocellular carcinoma cells (226). Overexpression of AIM2 inhibited breast cancer cell proliferation and induced apoptosis (227). AIM2 inhibits cell invasion and metastasis in RCC by enhancing autophagy-related gene expression (228). Chai et al. (229) constructed an AIM2-expressing adenovirus controlled by the CAIX promoter (Ad-CAIX-Promoter-AIM2). This virus significantly inhibited tumor cell proliferation and promoted apoptosis and cell killing by efficiently expressing E1A and AIM2 in renal carcinoma cells, further confirming the antitumor activity of AIM2 in RCC. Highly expressed SIRT1 in HPV-infected cervical cancer cells inhibited NF-κb-driven transcription of the AIM2 gene by destabilizing RELB mRNA and eliminated AIM2 inflammasome-mediated antiviral immunity, which allowed cervical cancer cells to continue to grow, suggesting that AIM2 exerts a tumor-suppressor role in cervical cancer (230).

Some evidence suggests that AIM2 has a protective function in CNS disease. Catherine R. Lammert et al. (231) found that defects in the AIM2 inflammasome affect brain development and the immune system’s ability to sense genotoxic stress. This leads to the accumulation of DNA damage in the brains of adult individuals, contributing to anxiety-related behaviors in mice. They further verified that AIM2 maintains neurological homeostasis through a specific regulatory effect on gasdermin-D, rather than through the inflammatory factors IL-1 or IL-18. The study by Ma et al. (232) that AIM2 negatively regulates the pathogenesis of experimental autoimmune encephalomyelitis (EAE) and that this effect is independent of the activation of inflammasome.AIM2 deficiency enhances the activation of microglia and infiltration of peripheral immune cells into the CNS, thereby promoting neuroinflammation and demyelination during EAE. Mechanistically, AIM2 plays a protective role in EAE by negatively regulating the DNA-PK-AKT3 pathway in microglia.

#### 3.5.5 Interaction of AIM2 with other cytoplasmic DNA sensors

AIM2 is one of the few cytoplasmic DNA sensors known to directly activate inflammasomes, with specific recognition of cytoplasmic dsDNA (whether from bacteria, viruses, or host cells) but not ssDNA or ssRNA, which makes AIM2 particularly important in the detection of DNA viral infections or damage to host DNA.AIM2 is found in a wide range of cell types expression, including immune cells (e.g. monocytes, T cells) and non-immune cells (e.g. epithelial cells). This wide range of expression allows AIM2 to function under a variety of physiological and pathological conditions.

There is synergistic activation of AIM2 with other cytoplasmic DNA sensors to produce immune amplification. Lee et al. found that (166), AIM2 can regulate the innate immune sensors Pyrin and ZBP1 to drive inflammatory signaling and trigger a form of inflammatory cell death known as PANoptosis, enhancing host defense. AIM2 acts synergistically with NLRP3. The study found that AIM2 forms a multiprotein complex with NLRP3, NLRC4, and Pyrin through interactions with ASC to induce PANoptosis in response to various inflammasome ligands (233). Therefore, AIM2 is a key driver of PANoptosis and may play a potential role in RA. Research by Walle et al. (234) indicated that A20 negatively regulates the activation of the NLRP3 inflammasome, reducing the production of IL-1β, thus providing a protective effect against arthritis, but it does not affect the activation of the AIM2 inflammasome. This suggests that there are still differences in the regulatory mechanisms between AIM2 and NLRP3. Additionally,AIM2 can be activated and amplified by IFN-I priming signals stimulated by cGAS in addition to being directly triggered by dsDNA.Xu et al. (235) developed a virus-like particle with high nucleic acid loading that induces both cGAS-STING activation and AIM2 inflammasome-mediated cellular pyroptosis to enhance anti-tumour immune responses.

Antagonism exists between AIM2 and IFI16, cGAS. IFI16 is also known to bind DNA and form inflammasome complexes, but IFI16 predominantly mediates the IFN-I response. Research has confirmed that IFI16 can heterodimerize with AIM2, negatively regulating the activation of Caspase-1 by the AIM2-ASC inflammasome (151). The AIM2 inflammasome inhibits cGAS-STING signalling. Corrales et al. (236) found that after exposure to cytoplasmic DNA, dendritic cells and macrophages lacking AIM2, ASC, or Caspase-1 exhibited cGAMP production, STING aggregation, and phosphorylation of TBK1 and IRF3. Additionally, there was an increase in IFN-β transcription. Mechanistically, AIM2 can attenuate the immune response activated by the cGAS-STING-TBK1-IRF3-IFN signalling pathway through competitive binding to dsDNA (237). AIM2 can inhibit TBK1 activation by competitively binding to dsDNA, in which the CARD structural domain of ASC can bind to the N-terminal transmembrane structural domain of STING or the CTT structural domain (238). The activated caspase-1 can directly bind to and cleave cGAS at the D^140/157^ site through its p20 domain, leading to a decrease in cGAMP levels (239). GSDMD inhibits the cGAS-dependent type I interferon response by triggering K^+^ efflux (240). AIM2’s inflammasome-independent function may also be involved, e.g. AIM2 can inhibit its own DNA sensing pathway by isolating the mouse homologue IFI205 from STING (241).

In addition, numerous studies have confirmed that IFN-I inhibits inflammasome activation such as NLRP1 and NLRP3 (242). However, there are no studies showing that IFN-I directly inhibits AIM2 inflammasome activation. Rheumatic diseases are either caused by inflammasome-induced pro-inflammatory secretion or by aberrant IFN-I production (243). AIM2 is known to be critical for regulating IFN-I responses, but how AIM2 is preferred over other DNA sensors remains to be elucidated. Together, these DNA sensing pathways constitute a fine-grained system of cellular recognition and response to cytoplasmic DNA, and the complex network of interactions and regulation that exists between them is a challenge for future research.

## 4 AIM2 and RA

Study identifies AIM2 gene as potential key gene associated with RA (244). This finding provides a new perspective for a deeper understanding of the pathogenesis of RA. Further, a study by Jing et al. (245) revealed that AIM2 is a common gene between RA and periodontitis (PD) based on crosstalk and cellular pyroptosis. Interestingly, the results of several epidemiologic studies have shown a significant correlation between PD and RA (246). Meta-analysis data by Qiao et al. also showed that PD patients had a 69% increased risk of RA compared to periodontally healthy individuals (247). All of them support that there may be common pathological mechanisms and molecular pathways between the two diseases, PD and RA, and AIM2 undoubtedly plays a pivotal role in these mechanisms.

AIM2 is associated with synovitis, vascular opacification formation, cartilage destruction, and bone erosion. Accumulation of cytoplasmic dsDNA is an important factor in FLS-mediated inflammatory response in rheumatoid synovitis (133). This phenomenon may trigger a series of inflammatory responses by activating the intracellular receptor AIM2, enhancing the recognition of dsDNA, thereby further promoting the inflammatory process in the synovium. DNase II predominantly digests dsDNA in the cytoplasm. In a mouse model of chronic polyarthritis induced by DNase II deficiency, arthritis-susceptible mice that were deficient in AIM2 exhibited significantly reduced signs of joint inflammation and associated histopathological manifestations (248). They also showed inhibition of synoviocyte hyperproliferation and immune cell infiltration, greatly reduced expression of MMP3, and attenuation of cartilage destruction and bone erosion (248).

The mRNA and protein levels of AIM2 and its downstream protein ASC were higher in RA synovium than in osteoarthritic (OA) synovium, while there was no significant difference in plasma (249). The expression of ASC was higher in serum of RA patients than in healthy subjects, in contrast to the expression of AIM2 in serum of RA patients, which was lower than that of healthy controls (249). The expression of these molecules was positively correlated with clinical features of RA, such as ESR and CRP levels, suggesting that AIM2 is involved in the inflammatory pathogenesis of RA (249). In addition, AIM2 is a member of the interferon-inducible (IFI) gene family, and IFN-γ induces the expression and activation of AIM2 inflammasome, induces the release of IL-1β, IL-18, TNF-α, and IL-6, and induces pyroptosis (182, 250). In RA patients, a decrease in AIM2-positive cells was observed, which was accompanied by a decrease in serum IFN-γ levels, and this decrease in AIM2 expression may be a consequence of lower IFN-γ levels (251). Moreover, IFN-γ may play a key role in RA to regulate immune response, reduce inflammation and prevent joint damage, although its exact mechanism of action still needs to be thoroughly studied and explored.

The inflammatory response mediated by FLS, along with angiogenesis and bone destruction, are important pathological processes in the formation of RA. The vicious cycle caused by the continuous activation of FLS to secrete inflammatory substances, which in turn promotes the inflammatory response, is a key reason for the exacerbation of RA (252). Silencing AIM2 in FLS by siRNA inhibits the proliferation but not the migration and apoptosis of RA-FLS, and thus inhibiting AIM2 may be an effective treatment for RA (249). Studies have shown that myricetin (MYR) significantly reduced the expression of the AIM2 gene and protein in RA-FLS (253). Both MYR treatment and AIM2 knockdown inhibited the proliferation of RA-FLS and reduced their migration and invasion (253). Additionally, AIM2 knockdown inhibited TNF-α-induced expression of IL-6, IL-8, CCL2, MMP-1, MMP-3, and MMP-13, which reduces TNF-α stimulation-induced AKT phosphorylation, thereby attenuating RA synovial inflammation and joint damage (253). Kassem et al. showed (254) that MYR has the ability to protect DNA from damage and cells from oxidative stress, so we hypothesized that the protective effect of MYR may reduce dsDNA in the cytoplasm thereby indirectly inhibiting the activation of AIM2 inflammasome. Experimental studies have shown that during AIM2 inflammasome activation, Caspase-1 activates multiple pathways that contribute to mitochondrial disassembly (255). This process increases mtROS, impairs mitochondrial membrane potential, and increases mitochondrial membrane permeability (255). Additionally, it amplifies mitochondrial dysfunction and promotes fragmentation of mitochondrial networks, ultimately enhancing inflammatory responses (255).

Unlike the other dsDNA sensors mentioned previously, the study by Mu et al. (256) employed various bioinformatics analysis methods and machine learning algorithms to identify AIM2 as a potential biomarker for RA, and it validated its diagnostic efficacy using an independent validation dataset. RA often causes atherosclerosis (AS), which is associated with a systemic inflammatory response and elevated dsDNA, in which inflammasomes and IL-1β are thought to be important inflammatory mediators, but the exact mechanism remains unclear (257). Impaired metabolic pathways of RA, production of free radicals, reduction of antioxidant systems, and release of pro-inflammatory cytokines trigger endothelial damage and atherosclerotic plaque susceptibility (258). The potential mechanisms of AIM2 inflammasome activation appear to involve increased oxidative glucose metabolism, mtROS generation, oxidative DNA damage, and DNA replication stress (259). These pathological processes may reinforce each other, forming a complex network that together drive the onset and progression of RA and AS. Study shows increased dsDNA deposition during advanced AS is accompanied by abundant AIM2 expression (260). Evidence suggests that AIM2 is a key protein mediating AS, capable of inducing inflammatory responses in arterial walls and promoting the formation of unstable atherosclerotic plaques (259). Changes in AIM2 levels may serve as a potential indicator of the risk of atherosclerosis associated with RA. Interestingly, it has been demonstrated that AIM2 can play an important role in suppressing T cells involved in autoimmunity by decreasing the AKT-mTOR signalling pathway and altering immune metabolism to enhance Treg cell stability (261). This indicates that the regulation of immune balance by AIM2 is crucial in RA, exerting both pro-inflammatory and anti-inflammatory effects. We cannot help but speculate that AIM2 may modulate the body’s immune tolerance to RA. Further research is needed to explore the specific mechanisms of AIM2’s role in RA and the potential conditions for its dual effects. Additionally, AIM2 has inflammasome-independent functions and may act as a rheostat in various inflammatory processes. For instance, AIM2 has been shown to bind to NETs, leading to the formation of DNAse-resistant nuclear protein fibers, serving as autoantigens in SLE (109).

## 5 Downstream effects mediated by the AIM2 inflammasome

The pathogenesis of RA, one of the most common systemic autoimmune diseases, has not been elucidated. However, cytokines are now found to perform many key biological processes, such as cell growth, proliferation, differentiation, inflammation, tissue repair, and regulation of immune response, and play an important role in the pathogenesis of RA (262). The overproduction and activation of inflammatory cytokines such as TNF-α, IL-1β, IL-6, and IL-18 cause inflammatory proliferation of synovial tissue, leading to degradation of cartilage tissue and destruction of bone tissue.

The secretion of pro-inflammatory cytokines IL-1β and IL-18 and the execution of cellular pyroptosis are the two main events that occur after activation of AIM2 inflammasome, both of which are closely related to the onset and progression of RA. Studies have confirmed that serum expression of IL-1β and IL-18 is significantly higher in RA patients than in the healthy population (263). The relative expression levels of localized AIM2 mRNA and the downstream protein IL-1β were also significantly higher in RA synovium than in OA (249). In a model of chronic polyarthritis triggered by its own dsDNA, a reduction in joint inflammation was associated with activation of caspases-1 and a significant reduction in the levels of the pro-inflammatory cytokines IL-1β and IL-18 in the diseased joints (248). The expression of AIM2 and its downstream molecule IL-1β can be inhibited by the application of AIM2 siRNA, which inhibits the proliferation of RA-FLS, showing that this pathway has an important role in the inflammatory response of RA (249).

### 5.1 IL-1β

In RA patients, elevated plasma and synovial fluid concentrations of IL-1 (IL-1α and IL-1β) correlate with various parameters of disease activity (264). IL-1β is produced mainly by monocytes and macrophages, but also by natural killer (NK) cells, T cells, B cells, endothelial cells, synoviocytes, and neutrophils, and it is a pleiotropic proinflammatory cytokine involved in a variety of autoimmune inflammatory responses (264, 265).

Synovial hyperplasia, pannus formation, and irreversible cartilage and bone destruction are the hallmarks of RA. IL-1β promotes the differentiation and proliferation of RA lymphocytes and synoviocytes. It stimulates the expression of phospholipase A2 (PLA2) and cyclooxygenase-2 (COX-2) in human synoviocytes, increasing their activity (266, 267). This process leads to the breakdown of lecithin membranes, producing arachidonic acid, which in turn results in the synthesis and release of prostaglandins (PGE2) in synoviocytes and chondrocytes, as well as collagenase synthesis and release in these cells (266, 267). PGE2 contributes to the pathophysiology of morning stiffness and arthralgia by sensitizing the body to injurious stimuli induced by bradykinin and histamine, as well as promoting plasma extravasation. This process enhances inflammatory pain and leads to edema formation (267). PGE2 binding to EP4 promotes the formation and activation of osteoclasts by enhancing RANKL expression (267). Additionally, it plays a role in inflammation-induced angiogenesis by stimulating the production of vascular endothelial growth factor (VEGF) (267).

The classical NF-κB pathway can be activated by various inflammatory cytokines, including IL-1β (268, 269). NF-κB expression and its binding to the receptor RANK are necessary for osteoclast formation, which enhances inflammatory bone loss (268, 269). IL-1β modulates leukocyte recruitment, matrix metalloproteinases (MMPs), and osteoclast activation via receptor activators of NF-κB ligand (RANKL), inducing cartilage degradation, bone erosion, and ultimately joint destruction (270–272). Cartilage is composed of proteoglycans and type II collagen, while tendons and bones are primarily made up of type I collagen (273). Collagenases MMP-1 and MMP-13 play significant roles in the destruction of rheumatoid joints (273). Notably, collagenase 3 (MMP-13), produced by chondrocytes, degrades type II cartilage collagen and proteoglycan molecules, demonstrating a dual role in matrix destruction (273). IL-1β induces fibroblast proliferation, which in turn induces synovial proliferation, and IL-1β can form a complex with HMGB1 to promote the secretion of pro-inflammatory cytokines by synovial fibroblasts and increase MMP-3 production (264, 274). In turn, MMP-3 degrades the non-collagenous matrix components of the joint.

### 5.2 IL-18

IL-18 expression is increased in the synovium and serum of RA patients, and there is a positive correlation between its increased levels and joint damage (275). The incidence and severity of collagen-induced arthritis was significantly reduced in mice lacking IL-18 (276). IL-18 promotes collagen-induced inflammatory arthritis through a mechanism that may be distinct from that induced by IL-12, with IL-18 treatment enhancing synovial proliferation, cellular infiltration, and cartilage erosion (277). IL-18 activates monocytes/macrophages and induces IFN-γ production, leading to an increased inflammatory response in RA, and inhibition of the IL-18 signaling pathway attenuates the RA inflammatory response (278).

IL-18 is an upstream cytokine of IL-1β, and in cartilage, IL-18 released from chondrocytes promotes cartilage degradation through a number of pathways through the (279). Macrophage colony-stimulating factor (M-CSF) and RANKL are both locally expressed in the synovial tissues of RA patients, and they are necessary and sufficient for the differentiation of precursor cells into mature osteoclasts (280). Osteoprotegerin (OPG) serves as a potent inhibitor of osteoclast maturation and activation both *in vivo* and ex vivo (280). Additionally, the RANKL/OPG ratio can be used to determine the extent of osteoclast-mediated bone resorption (280). It was demonstrated that IL-18 increased the expression of membrane-bound RANKL, the production of soluble RANKL, and M-CSF, as well as the RANKL/OPG ratio in RA FLS (280). This suggests that IL-18 may indirectly promote osteoclast differentiation through FLS in RA joints (280). Overall, the net effect of IL-18 on FLS facilitates the induction of osteoclast formation and bone resorption (280).

IL-18 also induces angiogenesis necessary to maintain the development of vascular opacities.SDF-1β/CXCL12 is a potent angiogenic factor that has been shown to induce angiogenesis in several *in vitro* and *in vivo* models (281, 282). VEGF is the most potent cytokine for inducing endothelial cell migration and proliferation, angiogenesis and increased capillary permeability (283). VEGF is involved in the development of RA synovitis. Studies have shown the presence of VEGF-positive cells in synovial tissues and high concentrations of VEGF in the synovial fluid of RA patients (283–285). Furthermore, serum VEGF concentrations were significantly higher in RA patients compared to those with OA, SLE, SS, and healthy controls (283–285). MCP-1/CCL 2 mediates angiogenesis through VEGF *in vitro* and *in vivo* (286). IL-18 increases the production of SDF-1β/CXCL12, MCP-1/CCL2 and VEGF through overlapping but distinct signaling pathways (287).

### 5.3 Pyroptosis

Activation of AIM2 inflammasome triggers pyroptosis, a pro-inflammatory programmed cell death regulated by the Gasdermin (GSDM) family, which is mainly characterized by membrane perforation, cell swelling and cell rupture and release of inflammatory factors (268, 269). Numerous studies have shown that cellular pyroptosis is involved in the pathogenesis of RA and that the incidence of cellular pyroptosis is positively correlated with RA disease activity. Wu et al. (288) found that compared to healthy controls, RA patients exhibited a significant increase in GSDMD expression and upregulation of LDH release in monocytes. Furthermore, typical cellular swelling and large vacuoles were observed under scanning electron microscopy, similar results were obtained when monocytes were cultured with serum from RA patients (288). Moreover, monocytes pretreated with RA serum with high disease activity (DAS28>5.1) showed higher expression of GSDMD, with enhanced cleavage of GSDMD proteins and release of IL-1β and IL-6, compared with RA serum with low disease activity (DAS28<2.6) (288). Compared with OA, RA synovial tissue showed lower pro-caspase-1 expression, higher caspase-1 expression, lower GSDMD expression and higher GSDMD-N expression (270). The expression level of the pyroptosis-associated marker protein GSDMD-N is significantly positively correlated with clinical indicators of RA disease activity, such as ESR, CRP, as well as the Krenn score of pathological synovitis and the subfraction of sublining inflammatory cell infiltration (270). The levels of LDH, IL-1β, and IL-18 in synovial fluid were significantly higher in RA patients than in OA patients and positively correlated with disease activity and inflammation (289). Furthermore, serum levels of IL-1β and IL- 18 were significantly higher than those in healthy controls (289).

Inhibition of cell pyroptosis can effectively inhibit the inflammatory response of RA joints, reduce the pathological changes of joints, and play a good RA prevention and treatment effect. Therefore, targeting and regulating cell pyroptosis will be an important means of prevention and treatment of RA in the future. Wu et al. demonstrated that amiloride can inhibit the pyroptosis of chondrocytes in AA rats through ASIC1a pathway, and morphologically improve the pathologic joint changes (including synovial hyperplasia, synovial thickening, pannus formation, and multiple inflammatory cell infiltration) in arthritic joints (290). Li et al. (291) found that Baihu Guizhi decoction (BHGZD) and its active components, mangiferin (MG) and cinnamic acid (CA), could inhibit GSDMD-mediated pyroptosis both *in vivo* and *in vitro*. They also observed a reduction in the activities of Caspase-1 and LDH, as well as a decrease in IL-1β and IL-18, which significantly improved the severity of AIA-M rats, reduced joint redness and swelling, decreased the incidence of arthritis, lowered the arthritis score and limb diameter, and increased the pain threshold. Ling et al. (292) found that the proportion of pyroptosis and LDH activity were reduced, and the expression of caspase-1/3/4/5 and GSDMD were decreased in mice treated with Jinwu Jianjian Bone Capsules (JWJGC)-containing serum, suggesting that JWJGC improves the tumor-like growth characteristics of RA-FLS by inhibiting cellular pyroptosis and suppressing its proliferation in RA treatment. Ge et al. found that punicalagin (PUN) attenuated the expression of inflammatory factors associated with cellular death and inhibited joint inflammation, cartilage damage and systemic bone loss in CIA mice (293). Mao et al. found that SMAD2 overexpression inhibited RA-FLS pyroptosis via the TGF-β pathway, and reduced the secretion of inflammatory cytokines such as IL-1β, IL-18, IL-6, IL-8, and LDH release, which in turn attenuated joint erythema, cartilage, and bone destruction in CIA rats (294). Chen et al. found that Hsa_circ_0044235 regulates RA pyroptosis through MiR-135b5p-SIRT1 axis, greatly reducing synovial proliferation, cartilage damage and inflammatory cell infiltration in CIA mice (295). Ren et al. showed that IL-37 could attenuate TNF-α-induced RA-FLS juxtaposition by inhibiting the NF-κB/GSDMD signaling pathway, reduce the expression of the inflammatory factors IL-18 and IL-1β in the cell supernatant, inhibit the proliferation of FLS, and attenuate the inflammatory response of RA joints, which serves as a therapeutic purpose for the prevention and treatment of RA (296).

## 6 Conclusion and prospect

RA is a chronic inflammatory disease with unclear etiology, which is prolonged, recurrent, and has a high disability rate, and may even be complicated by other systemic diseases, seriously jeopardizing the physical and mental health of patients. Although greater progress has been made in the treatment of RA in recent years, such as the clinical application of some disease-modifying anti-rheumatic drugs (DMARDs), including methotrexate, **leflunomide**, and TNF-α inhibitors, the treatment of RA still faces some challenges. Therefore, the development of new safe and effective treatments is crucial.

AIM2 inflammasome activation mediates cellular pyroptosis and releases pro-inflammatory cytokines IL-1β and IL-18, which are involved in the proliferation of synovial cells, the production of inflammatory mediators and the destruction of joint tissues in RA. However, there are fewer studies related to AIM2 inflammasome in the direction of RA, and the exact mechanism of its action in RA is still unclear. There are several targeted inhibitors of AIM2, including TTA GGG (297), J114 (298), comfreyin (Shikonin) (299) etc., but their efficacy and safety in treating RA are unknown and still need further research and validation.

In this paper, we review the sources of intracellular dsDNA that may activate AIM2, considering the latest research progress on AIM2. We also summarize the expression and function of other cytoplasmic DNA sensors, such as cGAS, IFI16, ZBP1, and TLR9 in RA. This review aims to provide new perspectives for future research and insights into the development of regulatory strategies targeting these sensors.AIM2 inflammasome appears to be a promising potential therapeutic target for RA and is expected to provide more theoretical basis for the diagnosis and treatment of RA with further research. Therefore the prevention and treatment of RA should emphasize the study of AIM2-related pathways.
